# Inter-hospital moderate and advanced Alzheimer's disease detection through convolutional neural networks

**DOI:** 10.1016/j.heliyon.2024.e26298

**Published:** 2024-02-15

**Authors:** Carlos Roncero-Parra, Alfonso Parreño-Torres, Roberto Sánchez-Reolid, Jorge Mateo-Sotos, Alejandro L. Borja

**Affiliations:** a Departamento de Sistemas Informáticos, Universidad de Castilla-La Mancha, Campus Universitario, Albacete, 02071, Spain; b Departamento de Ingeniería Eléctrica, Electrónica, Automática y Comunicaciones, Universidad de Castilla-La Mancha, Campus Universitario, Albacete, 02071, Spain; c Neurocognition and Emotion Unit, Instituto de Investigación en Informática, Universidad de Castilla-La Mancha, Campus Universitario, Albacete, 02071, Spain; d Instituto de Tecnología, Construcción y Telecomunicaciones, Universidad de Castilla-La Mancha, Campus Universitario, Cuenca, 16071, Spain

**Keywords:** Alzheimer, EGG, Deep learning, Convolutional neural network, Machine learning

## Abstract

Electroencephalography (EEG) has been a fundamental technique in the identification of health conditions since its discovery. This analysis specifically centers on machine learning (ML) and deep learning (DL) methodologies designed for the analysis of electroencephalogram (EEG) data to categorize individuals with Alzheimer's Disease (AD) into two groups: Moderate or Advanced Alzheimer's dementia.

Our study is based on a comprehensive database comprising 668 volunteers from 5 different hospitals, collected over a decade. This diverse dataset enables better training and validation of our results. Among the methods evaluated, the CNN (deep learning) approach outperformed others, achieving a remarkable classification accuracy of 97.45% for patients with Moderate Alzheimer's Dementia (ADM) and 97.03% for patients with Advanced Alzheimer's Dementia (ADA). Importantly, all the compared methods were rigorously assessed under identical conditions. The proposed DL model, specifically CNN, effectively extracts time domain features from EEG data in time, resulting in a significant reduction in learnable parameters and data redundancy.

## 1 Introduction

As of now, there are approximately 50 million individuals globally living with Alzheimer's disease (AD) [1], and he projected trend indicates that this number is expected to double every 5 years, reaching an estimated 152 million individuals with Alzheimer's disease (AD) by the year 2050. AD has an impact on the affected individuals, their families, and the economy, with estimated global costs of US$1 trillion each year. While there are therapies directed towards alleviating symptoms, it is crucial to highlight that, currently, there is no cure for Alzheimer's disease [2], [3].

The brain of a healthy adult is comprised of billions of neurons, each possessing extensive and branching extensions. These extensions play a crucial role in establishing connections between individual neurons, referred to as synapses. At these synapses, information is transmitted through small bursts of chemicals released by one neuron and absorbed by another. With trillions of synapses, the brain enables the rapid transmission of signals throughout its complex network. These signals form the cellular basis for memories, thoughts, sensations, emotions, movements, and skills.

Alzheimer's disease, characterized as a neurodegenerative disorder with a predominant impact on the elderly population, constitutes the primary etiology of dementia, accounting for 60-80% of documented cases. Diagnosis of AD typically relies on a composite assessment involving clinical evaluation, medical history, and cognitive testing. However, definitive diagnosis requires confirmation of the presence of beta-amyloid plaques and the formation of twisted strands of the tau protein inside neurons in post-mortem brain tissue samples [4]. These changes are accompanied by neuronal death and brain tissue damage. Inflamed condition and the atrophy of brain tissue represent additional associated alterations in Alzheimer's disease. Furthermore, a majority of individuals with AD experience one or more additional types of brain pathologies, commonly referred to as mixed dementia [5], [6]. Early symptoms may include difficulty remembering recent conversations, names, apathy, and depression. Communication problems, poor judgment, confusion, and behavioral changes may occur later. In the advanced stages of the disease, speaking, difficulty walking, and swallowing are common.

**Figure 1:**
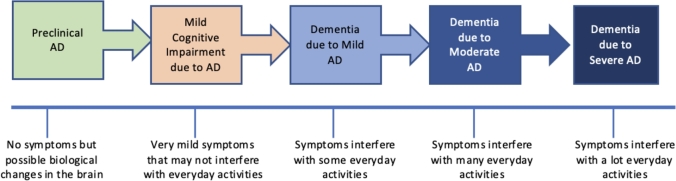
Alzheimer's disease (AD) spectrum.

The progression of AD results in brain changes that are not noticeable until memory loss becomes evident in the patient. This progression is characterized by three main phases: preclinical Alzheimer's disease (AD), mild cognitive impairment (MCI), and dementia, also known as Alzheimer's dementia (Fig. 1) [7], [8]. The phase of Alzheimer's dementia is additionally segmented into mild, moderate, and advanced stages of dementia. It is acknowledged that the progressive development of Alzheimer's disease commences with preclinical Alzheimer's disease (characterized by the absence of symptoms) and culminates in severe or advanced Alzheimer's dementia, marked by pronounced and debilitating symptoms. However, the duration of each stage is not uniform and fluctuates depending on patient characteristics such as age, genetics, biological sex, and other factors. [9].

As AD advances, individuals typically manifest a spectrum of evolving symptoms that are indicative of varying degrees of neuronal damage across distinct brain regions. The progression of dementia symptoms, transitioning from mild to moderate, and ultimately severe, exhibits considerable heterogeneity among individuals. In the mild stage of Alzheimer's dementia, individuals often retain a degree of independence in numerous aspects but may necessitate assistance with certain activities to optimize their autonomy and safety. Challenges may arise in managing financial affairs and attending to bills, and there might be a prolonged duration required for the completion of routine daily tasks. Although individuals in this stage may still be capable of driving, working, and participating in favored activities, cognitive difficulties may become increasingly apparent [10]. Moderate Alzheimer's Dementia (ADM): The moderate phase of Alzheimer's dementia, frequently characterized as the lengthiest stage, is marked by an exacerbation of memory and language difficulties. Individuals in this stage are more prone to confusion and encounter heightened challenges in executing multi-step tasks, such as bathing and dressing. Incontinence may manifest intermittently, accompanied by notable personality and behavioral alterations, including heightened suspicion and agitation. Recognition difficulties, encompassing challenges in identifying loved ones, become more pronounced during this phase [10]. Advanced Alzheimer's Dementia (ADA): During the severe stage of Alzheimer's, individuals undergo a substantial decline in verbal communication abilities, requiring continuous care. The impairment in brain regions controlling movement confines patients to bed, exposing them to increased risks of physical complications, including blood clots, skin infections, and sepsis, initiating systemic inflammation that can result in organ failure. The damage to cerebral regions governing swallowing worsens challenges in eating and drinking, potentially leading to food aspiration into the trachea instead of the esophagus. Consequently, food particles may be deposited in the lungs, triggering lung infections. This type of infection, known as aspiration pneumonia, serves as a contributing factor to the mortality of numerous Alzheimer's patients [11].

In this study, patients with ADA and ADM are analyzed, as these are the most aggressive stages of the disease. Early detection of these stages would provide time to effectively treat the disease.

• Using the United States as a benchmark for acquiring an objective statistical sample, it becomes apparent that approximately 6.7 million individuals are afflicted by Alzheimer's disease (AD). Within this population, 26.7% fall within the age range of 65-75 years, 37.9% are between 75-84 years old, and 35.4% are aged 85 years and older [12]. The initial inference derived from this data is that the probability of developing Alzheimer's dementia rises with age, and moreover, it stands as a prominent cause of death in individuals aged over 85 years. According to recent studies [10], it is estimated that 13.8 million people will suffer from Alzheimer's dementia by 2060, indicating a significant increase compared to current data. Analyzing the number of deaths per 100,000 population caused by this disease, it has increased from 17.6% in 2000 to 37% in 2019, representing an increase of more than double in mortality over a 20-year period [13], [14].
• Presently, Alzheimer's disease (AD) diagnosis involves standardized mental status tests, frequently necessitating costly neuroimaging scans and invasive laboratory procedures, leading to a protracted and expensive diagnostic protocol. Nevertheless, in the past decade, electroencephalography (EEG) has emerged as a non-invasive alternative for investigating Alzheimer's disease, offering competition to more expensive neuroimaging techniques like magnetic resonance imaging (MRI) and positron emission tomography (PET) [15]. EEG offers several advantages over other techniques for studying brain function, including its affordability, ability to tolerate subject motion, and absence of radiation exposure risks. However, it also has some drawbacks, such as limited spatial resolution and a relatively poor signal-to-noise ratio. EEG is particularly suitable for analyzing Alzheimer's disease in early stages, which often progress to more aggressive stages of the disease [16]. Almost all hospitals have EEG equipment, making this acquisition procedure widely available. Additionally, its usage and handling are straightforward for any physician, making it easily accessible and manageable.
• Artificial intelligence, whether employing machine learning (ML) or deep learning (DL) methodologies, relies on a set of mathematical models and algorithms to iteratively enhance the performance of specific tasks. The process commences with a training dataset as input, guiding estimations without the need for explicit programming. Tasks within this domain exhibit a broad spectrum and can be broadly categorized into two groups: supervised and unsupervised learning. Both modes involve learning from datasets with provided inputs and known outputs. Supervised learning constructs a predictive model using both input and corresponding output data, falling into the subcategories of classification and regression, generating discrete and continuous outcomes, respectively. On the other hand, unsupervised learning constructs a predictive model using only input data, further categorized into clustering and dimensionality reduction, producing discrete and continuous outcomes, respectively [17].
• Various machine learning models can be applied to EEG device data. Classification: These are algorithms that assign labels or categories to a set of data based on their features using the input data used in training. The goal is to train a classification model to accurately predict the class or category to which previously unseen input data belongs. These methods prove highly effective in categorizing distinct mental states, such as sleep and wakefulness, or different types of brain activity, such as alpha and beta waves, when applied to EEG device data. Among the commonly utilized classification algorithms for this purpose are Support Vector Machine (SVM), Bayesian Linear Discriminant Analysis (BLDA), Decision Trees (DT), and Gaussian Naive Bayes (GNB) [18]. Regression: Regression modeling is a widely employed statistical tool due to its simplicity in establishing functional relationships between variables. In the context of analyzing data from brain electrical activity, regression models prove valuable for predicting future values. For instance, a regression model can forecast an individual's stress level based on their brain activity at a specific moment. Among the frequently utilized regression algorithms for such purposes are k-Nearest Neighbors (KNN), Random Forest (RF), and eXtreme Gradient Boosting (XGB) [18]. Clustering: These are models that group a set of data into clusters, so that objects within the same cluster are similar to each other. Utilizing a clustering model allows the grouping of individuals based on their patterns of brain activity, revealing similarities or differences among them. Commonly employed clustering algorithms for such applications include K-means, k-Medoids, Self-Organizing Map, Fuzzy c-Means, Gaussian Mixture Hierarchical Clustering, and DBSCAN [18]. Dimensionality Reduction: These are techniques and methods used to decrease the number of variables or features in a dataset. The objective of dimensionality reduction is to simplify the data representation while preserving as much relevant information as possible. These methods are employed when dealing with large datasets, many of which may contain irrelevant or redundant information, in order to reduce model complexity. Principal Component Analysis (PCA), Linear Discriminant Analysis (LDA), Singular Value Decomposition (SVD), and t-Distributed Stochastic Neighbor Embedding (t-SNE) are some commonly used methods in the field of artificial intelligence [19].
• Machine learning models can undergo training and fine-tuning through approaches such as supervised learning and unsupervised learning. Following the training process, these models can be employed to analyze real-time data, providing accurate and comprehensive insights into brain activity [20]. In the coming years, ML algorithms that have undergone could surpass human decision-making in classification performance. In this case, they could act as autonomous agents with quality control monitoring by humans [21].

This study utilizes deep learning algorithms, a subset of machine learning that centers on the application of deep artificial neural networks (ANN), for the classification of EEG data. Comprising multiple layers of interconnected nodes termed “neurons,” these networks process and transmit information. Within an artificial neural network (ANN), the intermediate layers, referred to as hidden layers, adeptly acquire intricate and abstract representations of the data as information is propagated through the network. In summary, artificial neural networks (ANNs) possess the capacity to learn intricate patterns in the data, delivering highly accurate performance owing to their capability to acquire abstract representations of the data. Some frequently employed deep learning techniques include Recurrent Neural Networks (RNN), Radial Basis Function Networks (RBF), Convolution Neural Netwotks (CNN), Long Short-Term Memory (LSTM), Side-Output Residual Networks (SRN), and Gated Recurrent Units (GRU) [22], [23].

The current investigation enables the enhancement and fine-tuning of Alzheimer's disease detection in patients at medium to advanced stages of the condition through the utilization of Convolutional Neural Network (CNN). It is a model where neurons are organized into layers and specialize in detecting specific features such as shapes, edges, and textures. In general, this architecture consists of multiple layers whose function is to extract features from the input data. In this instance, data sourced from various medical centers, along with a population sample exceeding 650 patients from five distinct hospitals, has been employed. This utilization has resulted in the creation of an output data set referred to as a feature map (feature mapping). This is achieved by applying the mathematical operation called convolution. Subsequently, before performing another convolution, it is necessary to reduce the number of neurons, as otherwise, its number would be too high in the next layer. This is done through a process called pooling, which reduces the dimensionality of the extracted features while retaining the most important ones. Ultimately, following successive convolutions and identification of essential patterns, the final layer establishes a connection with a fully connected layer, where an activation function is applied. Subsequently, this connects to an output layer containing neurons corresponding to the classes undergoing classification.

The article is structured as follows: Section 2 provides an overview of the materials utilized in this study. Section 3 details the proposed classification approach, the description of the proposed network, and the employed validation method. Results and their discussion are presented in Sections 4 and 5, respectively. Ultimately, Section 6 summarizes the conclusions drawn from this work.

## 2 Equipment and materials

Electroencephalography (EEG) is a technique that was first used in 1928 but has evolved over the years to enable the digitalization of brain data for subsequent analysis [24]. Despite significant technological advancements, there are still challenges in capturing data from the patient's scalp, requiring qualified clinical personnel to perform the procedure. Nevertheless, EEG can be considered an economical, non-invasive method that does not pose radiation risks and is available in almost all hospitals worldwide [15]. Additionally, the expertise of the clinician can help to reduce noise caused by patient movement, breathing, and sweating. In this particular case, highly specialized professionals conducted sampling at 1000 Hz and utilized filtering techniques [25], incorporating a 50 Hz notch filter along with a low-pass filter covering a frequency range from 0.5 Hz to 70 Hz.

**Figure 2:**
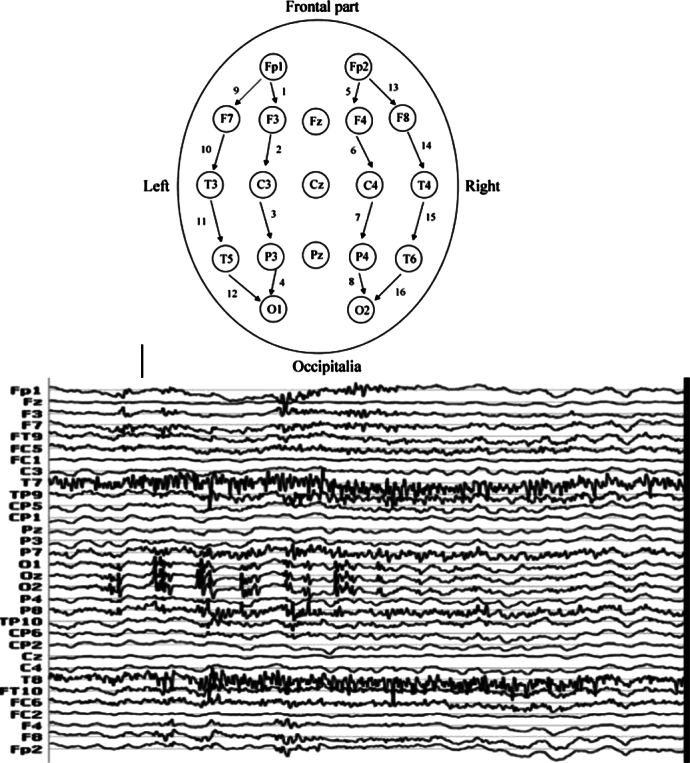
Electroencephalography (EEG). 32 electrodes of the EEG channels and EEG signals of 32 channels [27].

Continuous EEG recordings were obtained using a 32-channel brain imaging system utilizing synthesized AG/AgCl electrodes, as shown in Fig. 2. To capture and store the brain's electrical activity, the participant wore an EEG cap on the top of their head, necessitating consistent spatial positioning of the electrodes on the scalp in accordance with the international 10-20 system [26] with reference points Fpz/Afz/Fz/Cz/P/Qz. This setup allowed for recording the electrical activity in the form of waves, at a rate of 1000 Hz sampling frequency. Technically, a 32-channel encephalogram functions by placing 32 electrodes on the patient's head. These electrodes are connected to a recording system that stores the brain's electrical activity, which is then presented graphically as an EEG trace.

In this context, numerous signal processing methods have been suggested to isolate and extract meaningful features that assist in diagnosing complex characteristics that may arise. While EEG exhibits similarities across all hospital centers, the proficiency and specialization of the clinicians conducting the tests, incorporating patients from diverse hospitals, contribute to more accurate and dynamic statistical data in the development of results, rendering them more robust.

The conducted study carries significant importance owing to the compilation of data from various Spanish hospitals, contributing to diversity and augmenting statistical reliability. The selected hospitals include Hospital General Universitario in Valencia, Hospital Virgen de la Luz in Cuenca, Hospital Universitario Virgen de las Nieves in Granada, Hospital Clínico Universitario in Salamanca and Hospital Río Hortega in Valladolid.

To execute this investigation and execute diverse computations, a computing system featuring an Intel Xeon dual-core processor and 32 GB RAM was employed. The MATLAB software served as the computational tool for processing the electroencephalogram (EEG) data acquired from patients across various scrutinized hospitals.

Demographic data spanning from 2011 to 2022 for the examined patients and controls are delineated in Table 1. Specifically, EEG signals from 668 participants underwent scrutiny, with a breakdown of 261 individuals in the control group, 201 diagnosed with moderate Alzheimer's disease, and 206 diagnosed with advanced Alzheimer's disease. The mean age for each group was 70.4, 71.3, and 72.6 years, respectively. Additionally, the average educational level among participants was 8.5, 5.1, and 4.9, respectively.

**Table 1:**
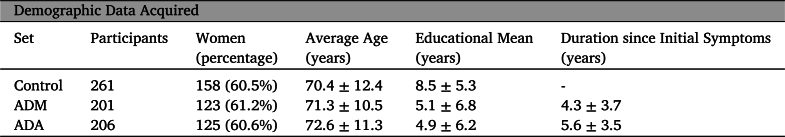
Demographic data acquired.

## 3 Methodology

Acknowledging the success of machine learning approaches in various domains, such as epidemic control [28] and medical image classification [29], and considering the substantial volumes of data within the healthcare domain, it is reasonable to employ ML/DL methods in medicine for supporting disease classification and diagnosis. [21].

An Artificial Neural Network (ANN) operates as a computational model, deriving its functions from biological neural networks and taking inspiration from the observed behavior of the human brain. These systems manifest non-linear characteristics and facilitate adaptation to various objectives. Their architecture typically includes an input layer, multiple hidden layers consisting of neurons, and an output layer. The input layer receives stimuli for the artificial neuron, the hidden layers process these stimuli, and the outputs represent the responses to the input stimuli. Neurons in the hidden layers can adapt and learn by adjusting their synaptic weights, allowing them to be modified to achieve a specific objective [18], [30]. It has been demonstrated that using Artificial Neural Network and Convolutional Neural Networks (CNN) for dementia detection results in high accuracy and reduced execution time [31].

### 3.1 Suggested CNN architecture

**Figure 3:**
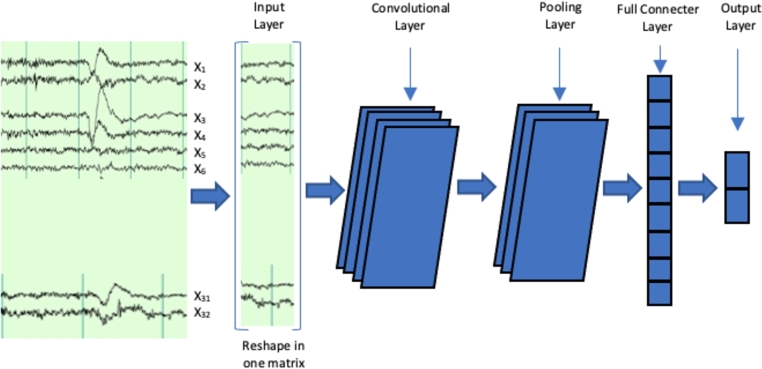
Proposed CNN neural network structure.

The recommended CNN network can be used to classify different multichannel EEG signal samples for class detection. The CNN model is shown in Fig. 3. In total, there are 6 layers in the proposed DL model using the CNN architecture, consisting of 3 convolutional layers, one flattening layer, and two fully connected (FC) layers [32]. The DL model has been constructed in a 2D matrix form to accommodate the EEG data, where the input data includes quantitative variables as a function of time (in particular, a 2D matrix measuring the time and amplitude of EEG signals from 32 channels), resulting in class-based outputs (ADA or ADM). In addition, Batch normalization is implemented after each convolutional layer. Gaussian dropout weights were also used in this CNN model. This is because transfer learning protocols suggest starting with random Gaussian distributions while learning the CNN from scratch.

Transfer learning can be used by loading pre-trained weights to improve network initialization. It can inherit the features of the trained network and improve training efficiency. Next, Adam optimization has been set to 0.0001 with 200 epochs [33].

There are three classes in this classification. The output layer of the CNN consists of 2 classes for detecting control and ADA patients, as well as control and ADM patients. The input consists of a 2D matrix, which corresponds to the time-domain field and their corresponding amplitude captured with the EEG electrodes. The structure is captured in the 32 input channels of the EEG, resulting in 32 matrices per analyzed patient.

The input layer initiates the process by delivering the feature matrix, which is scrutinized by a convolutional layer and subjected to max pooling. Following this, the remaining three fully connected (FC) layers employ a dropout of 0.8. The detailed model of the CNN architecture is illustrated in Fig. 3. The sets of hyperparameters can be seen in Table 2. In the particular case of the proposed CNN the batch size of the convolutional layers (conv2d) is 512, the conv2d model's mean squared error (MSE) is defined by a minimized function, utilizing a stride of 1 for both conv2d and max pooling layers. The optimizer component is configured to employ the Adam optimizer.

**Table 2:** Primary hyperparameters of the machine learning algorithms assessed in the investigation.

Machine Learning Method	Hyperparameters
SVM	*Kernel function:* Gaussian
	*Iteration limit* = 100
	*Sigma* = Gaussian
	*Numerical tolerance* = 0.001
	*C* = 1

DT	*Maximum depth* = 100
	*Minimum instances in leaves* = 4
	*Minimum instances in internal nodes* = 6

KNN	*Number of neighbors* = 20
	*Weight:* Uniform
	*Distance metric:* Euclidean

RNN	*Learning Rate:*0.005
	*Max number of neurons:* 150
	*Iteration limit:* 500
	*Number of fully connected layers:* 3

CNN (our study)	*Adam optimization:* 200 epochs
	*learning rate:* 0,0001
	*Number of Layers:* 23 in 3 modules.
	*Strides:* 2
	*Fully Connected Layers:* 4 FC
	*Batch Size:* 512
	*Pooling Size:* 160 (5 scales / 32 features)
	*Learning Rates:* 10^−3^

This research work collected 668 patients, using 17,100 labeled matrices, and 70% of the subjects were used to train the CNN. The 5-fold cross-validation technique was applied [34]. With the increase in network complexity, the machine typically learns more, but deeper networks will extend the processing time. To avoid overfitting in this study, cross-validation techniques have been employed, enhancing the generalization capability of the CNN network. In the analysis, building a network with a fast training period was a main priority.

Finally, feature extraction serves as a pivotal process, where the primary objective is to discern and select the most pertinent attributes from EEG data. This serves a dual purpose: it reduces data dimensionality, consequently enhancing model performance, and it augments the interpretability of the data. Subsequently, these extracted features become instrumental in training classification models. In our study, various features have been employed for machine learning classification methods i.e., SVM, DT, and KNN. More precisely, the analysis involved the application of Detrended Fluctuation Analysis (DFA), Approximate Entropy (ApEn), Hurst exponent (HE), Higuchi fractal dimension (HFD), Lyapunov exponent (LE), and EEG band power. Notably, ApEn and EEG band power demonstrated a reduced computational load compared to the remaining techniques. This optimization facilitated the rapid computation of these features, enabling nearly real-time interpretation of the analyzed EEG data for each patient. Remarkably, even in our particular scenario, the computational analysis of each patient's data was efficiently completed within seconds.

On the contrary, it is crucial to emphasize that within the domain of deep learning, particularly with algorithms such as Recurrent Neural Networks (RNN) and Convolutional Neural Networks (CNN), conventional feature extraction methods are eschewed. These neural networks possess an inherent ability to autonomously capture and extract the fundamental properties or features from the input signal as it traverses through the diverse layers of their architecture.

### 3.2 Evaluating the proposed convolutional neural network (CNN) model

**Figure 4:**
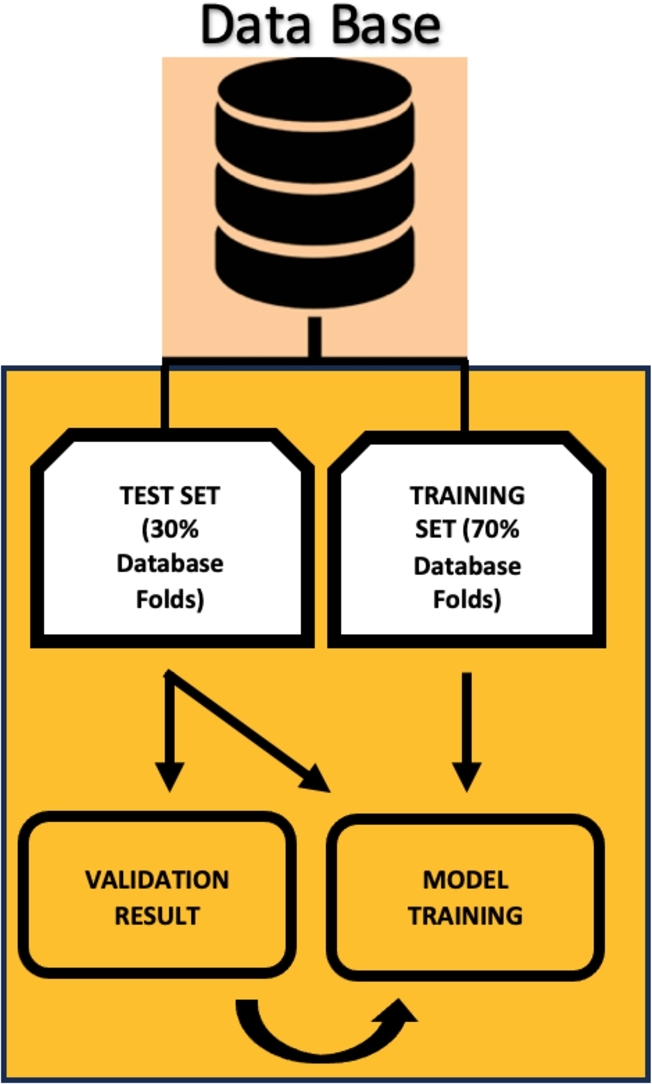
Validation method used.

In this research, the input dataset was partitioned, allocating 70% for training and 30% for testing and validation. This partitioning aimed to mitigate overfitting, and therefore, cross-validation was executed without intermixing the designated data. The proposed model underwent validation through a K-fold cross-validation procedure [35], enabling the assessment of predictive capabilities. Fig. 4 visually represents the data selection process employed in the study and the acquisition of the trained model.

In algorithmic evaluation, meticulous tuning of diverse parameters, denoted as hyperparameters, is imperative for the purpose of modifying and optimizing outcomes. These hyperparameters encompass variables such as the count of divisions, layers, kernel functions, learners, neighbors, neurons, among others. The goal of this experimentation is to enhance performance via Bayesian optimization by fine-tuning hyperparameters, selecting values that produce optimal results as evaluated by metrics such as the area under the curve (AUC) and balanced accuracy. In this context, a comprehensive analysis of the crucial hyperparameters related to the machine learning algorithms investigated in the study is presented in Table 2.

## 4 Outcomes

In this section, the outcomes and statistical analysis of our experiments are showcased. The performance of each experiment was documented using various performance metrics i.e., Negative Predictive Value, Balanced Accuracy, Specificity,F1, Sensitivity Recall, Precision, Specificity, AUC, MCC, Kappa and DYI. To assess the proposed Convolutional Neural Network (CNN), the obtained results are compared against four other machine learning algorithms and another deep learning classifier. This comparison involves a binary classification task, specifically categorizing patients with Advanced Alzheimer's Disease (ADA) or Moderate Alzheimer's Disease (ADM) concerning a control group.

The results of these experiments can be observed in Table 3, Table 4, where different DL and ML algorithms are compared. In this case, the SVM, DT, KNN, RNN, and CNN algorithms have been analyzed. It is evident that the proposed CNN method attains the highest performance, outperforming the other analyzed algorithms and achieving a balanced accuracy of 97.45% for Advanced Alzheimer's Disease (ADA) and 97.03% for Moderate Alzheimer's Disease (ADM). Additionally, it can be observed the notable improvement obtained for the proposed algorithm based on CNNs compared to ML algorithms, with the SVM algorithm performing poorly, achieving a balanced accuracy of only 89.26% for ADA and 89.61% for ADM. Additionally, no significant differences were observed during the classification of patients with Advanced Alzheimer's Disease (ADA) or Moderate Alzheimer's Disease (ADM).

**Table 3:** Metrics of performance for ADA classification using methods of ML/DL. All values are represented in percentages %.

ML/DL Method	Balanced	Sensitivity Recall	Specificity	Precision	Negative Predictive	AUC	F1	MCC	DYI	Kappa
	Accuracy				Value					
SVM	89.26	89.37	89.16	88.62	88.42	89	88.99	79.2	89.26	79.46
DT	88.52	88.63	88.42	87.89	87.68	88	88.26	78.55	88.52	78.81
KNN	90.78	90.89	90.67	90.13	89.92	90	90.51	80.55	90.78	80.82
RNN	90,53	90,64	90,42	89,88	89,67	90	90,26	80,33	90,53	80,59
CNN	97,45	97,57	97,34	97,76	96,54	97	97,16	86,58	97,45	86,87

**Table 4:** Metrics of performance for ADM classification using methods of ML/DL. All values are represented in percentages %.

ML/DL Method	Balanced	Sensitivity Recall	Specificity	Precision	Negative Predictive	AUC	F1	MCC	DYI	Kappa
	Accuracy				Value					
SVM	89.61	89.72	89.5	88.97	88.76	89	89.34	79.51	89.61	79.78
DT	88.16	88.27	88.06	87.53	87.33	88	87.91	78.23	88.16	78.49
KNN	90.36	90.47	90.25	89.71	89.54	90	90.09	80.18	90.36	80.44
RNN	90,14	90,25	90,03	89,5	89,29	90	89,87	79,98	90,14	80,25
CNN	97,03	97,12	96,89	96,32	96,09	97	96,71	86,18	96,12	86,47

**Figure 5:**
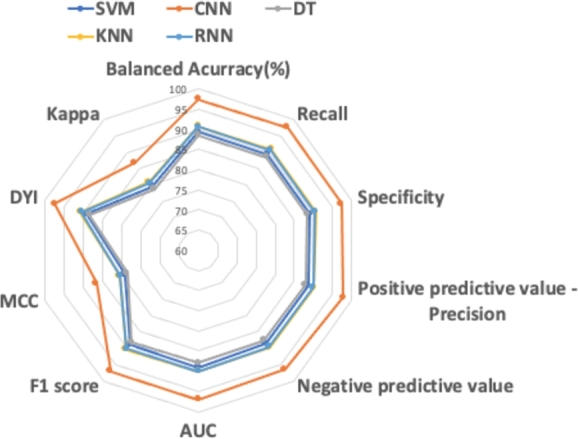
Radarplot depicting the performance of the algorithms used in the study for patient classification in ADA.

**Figure 6:**
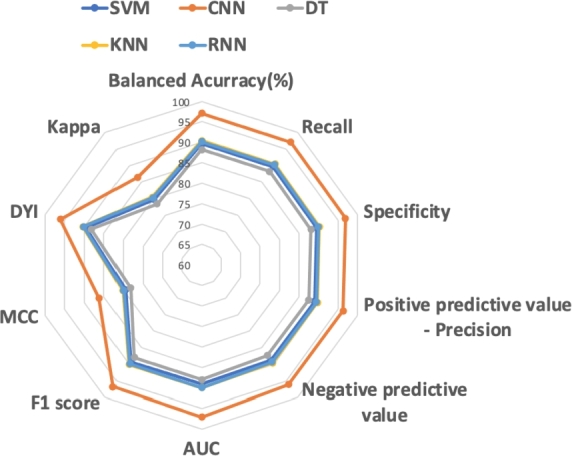
Radarplot depicting the performance of the algorithms used in the study for patient classification in ADM.

Two radar charts, illustrated in Figure 5, Figure 6, visually depict the performance metrics of each Machine Learning (ML) and Deep Learning (DL) method assessed. The CNN model exhibits the most optimal shape, signifying superior classification performance for Advanced Alzheimer's Disease (ADA) and Moderate Alzheimer's Disease (ADM) compared to the control subjects. These results are aligned with the findings presented in Table 3, Table 4.

**Figure 7:**
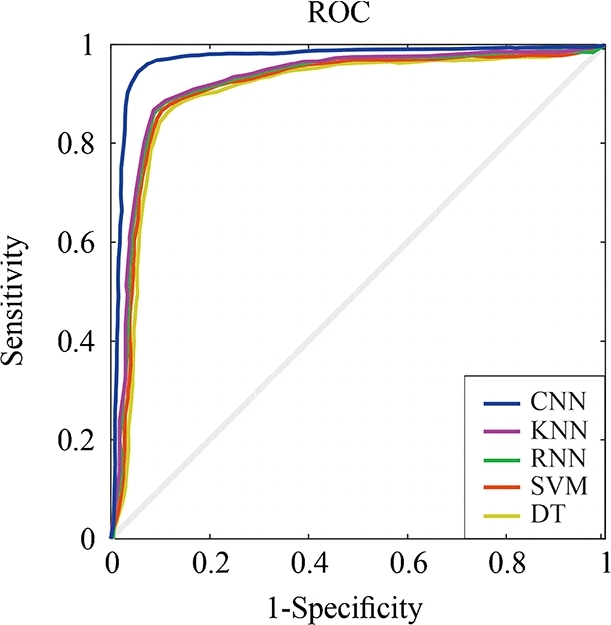
The ROC curve depicting the classification of Advanced Alzheimer's Disease (ADA) through both ML and DL methods.

**Figure 8:**
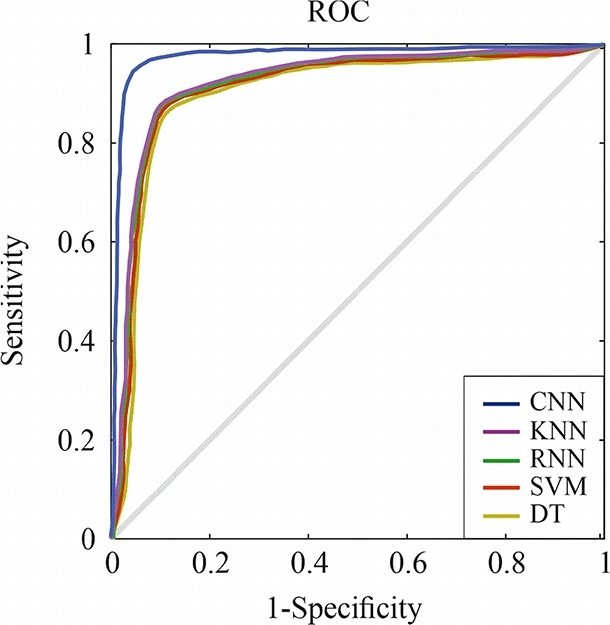
The ROC curve depicting the classification of Advanced Alzheimer's Disease (ADM) through both ML and DL methods.

The accuracy of the experiments has been also evaluated derived from the results provided by the Receiver Operating Characteristic (ROC) curves. These curves assess the ability of a classification model or algorithm to distinguish between positive and negative classes. They depict the connection between sensitivity (True Positive Rate: TPR) and specificity (False Positive Rate: FPR) across different decision thresholds. The closer the ROC curve is to an Area Under the Curve (AUC) of 1, the better the model's performance in terms of precision and ability to accurately discriminate between classes. In our case, the CNN algorithm approaches a value of 1, indicating excellent and almost perfect results for both ADA and ADM patient classification, as seen in Figure 7, Figure 8. It can be also observed that the other set of analyzed algorithms have significantly lower specificity compared to the CNN algorithm, suggesting that this configuration and method achieve nearly perfect results.

## 5 Discussion

**Figure 9:**
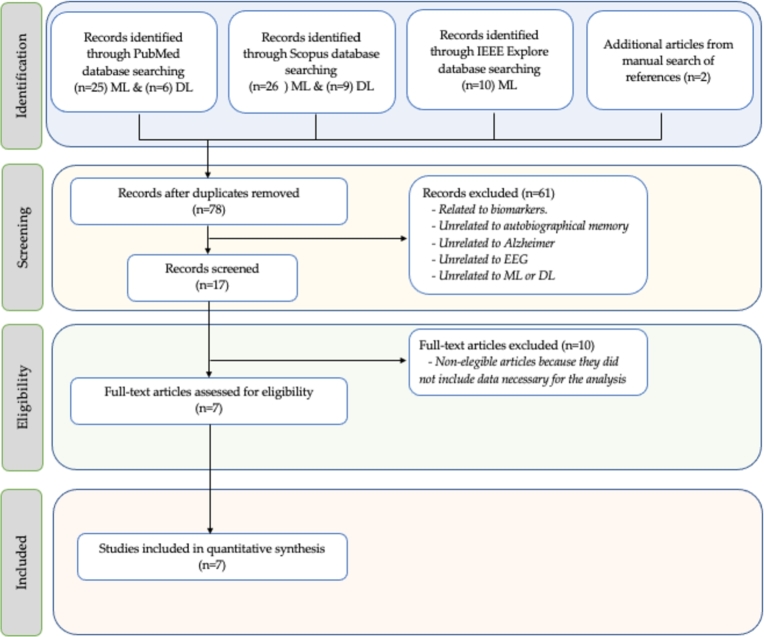
The workflow of the PRISMA methodology.

A search of the last published works in numerous scientific journals, which have analyzed the use of ML/DL algorithms for classifying patients with ADA/ADM, has been first conducted to demonstrate the advantages of the proposed classification network. To carry out this study, the Preferred Reporting Items for Systematic Reviews and Meta-Analyses (PRISMA) analysis has been chosen as the framework for conducting the systematic review and meta-analysis in this scientific research, as it can be seen in Fig. 9. This analysis aims to enhance the transparency, quality, and integrity of the conducted study [36]. It will encompass four phases: identification, selection, eligibility, and inclusion.

Initially, sources or databases for retrieving the most relevant articles worldwide on this topic were chosen, including IEEEXPLORE, PubMed, Scopus, PsycINFO, and manual searches. The search terms employed were (“Alzheimer” AND “EEG” AND “Deep learning” AND “Resting state”) or (“Alzheimer” AND “EEG” AND “Machine learning”). Only peer-reviewed original articles in English were considered, and records from 2019 to June 2023 were examined to focus on the most recent studies. A total of 78 records were identified through the database search. After removing duplicates, biomarker-related or dementia-related articles, articles unrelated to ML/DL methods, articles not involving EEG signal capture, and articles not exclusively focused on Alzheimer's disease, 7 articles were selected for review.

Table 5 provides a comparison between the final selected articles and the Convolutional Neural Network. It encompasses accuracy for Advanced Alzheimer's Disease (ADA) and Moderate Alzheimer's Disease (ADM), dataset size, feature extraction technique, and the algorithms utilized for classification.

**Table 5:** Contrast with prior studies.

Reference	C vs. ADA Precision	C vs. ADM Precision	Database Size	Characteristic extraction	Method
[31]		96,2	40	-	CNN
[37]	92,7	95,6	54	DFT/FFT	Conv-LSTM / Conv-BLSTM
[38]		97,10	49	-	CNN
[39]	88,0	-	26	HC / SE / RP	SVM
[40]	-	89	54	-	CNN
[41]	99,98	99,98	86	DWT technique	KNN
		99,7	86	DWT technique	SVM
[42]	87,9	-	23	ERP	SVM
Our study	97,49	97,03	668	-	CNN

Upon analyzing the data extracted from various articles pertinent to our study, several significant observations have come to light. Firstly, it is evident that the databases and the number of subjects studied in these articles are notably limited. This inherent limitation could potentially result in overfitting in specific instances. Furthermore, it is worth noting that there is a dearth of individual studies that concentrate on patients with ADA and ADM separately.

In terms of the metrics aspects of our study, our CNN algorithm has demonstrated commendable accuracy results, achieving 97.49% for ADA and 97.03% for ADM. This accomplishment becomes even more noteworthy when considering the dataset size employed in our research, which encompasses 668 subjects. This figure stands out remarkably as it is 7 to 29 times larger than the sample sizes utilized in prior investigations. Consequently, it is reasonable to conclude that our study exhibits a superior level of representativeness in its results.

Nevertheless, when research embarks on the endeavor of comparing findings with those of previous studies, it becomes apparent that such a task is fraught with complexity and carries the risk of leading to erroneous conclusions. The primary reason for this complexity is the absence of a standardized methodology across studies, particularly concerning preprocessing techniques, feature extraction methods, and dataset sizes. It is crucial to emphasize that the results attained in this study were achieved without employing any specific feature extraction method and benefited from a significantly larger dataset compared to those referenced in the literature, as previously mentioned.

Furthermore, our exploration did not uncover any articles that incorporated data from multiple distinct hospitals. It is pertinent to highlight employing data from a singular source, whether it be a single hospital or geographic area, tends to diminish the diversity and representativeness of the study results, even if it results in higher accuracy.

The principal contributions of this research can be summarized in the comprehensive algorithm comparison presented in the Table 3, Table 4. This comparison stands out, given that it employs the same dataset and optimization procedures consistently across all ML and DL methods. This ensures a fair and equitable basis for comparison. The superior accuracy demonstrated by the CNN algorithm in this context holds the potential for profound implications in various classification tasks. Our proposed deep learning model, which harnesses a 6-layer CNN architecture, has facilitated rapid training and classification.

A significant innovation of our study lies in the development of a deep learning model that automatically extracts time-domain features from EEG data. This approach effectively reduces the number of learnable parameters and eliminates data redundancy, ultimately enhancing algorithmic efficiency. It is also imperative to highlight that our study encompasses a notably larger database compared to any previous investigations, incorporating data from five different hospitals and various healthcare professionals involved in patient assessments. This multi-hospital and multi-professional approach substantiate the robustness and precision of our presented results.

Finally, our deep learning model, tailored to classify Control vs. ADA and Control vs. ADM, has yielded remarkable accuracy rates of 97.49% for ADA and 97.03% for ADM. To our knowledge, our study represents the initial endeavor to concurrently classify Alzheimer's disease in patients with Advanced Alzheimer's Dementia (ADA) and Advanced Mild Dementia (ADM) using CNN neural networks. The model crafted in this research demonstrates considerable potential for integration into brain activity analysis systems, aiming to predict Alzheimer's disease in patients.

## 6 Conclusions

The proposed CNN-based model represents a noteworthy leap in the field of Alzheimer's disease detection utilizing EEG recorded samples. This model is constructed with meticulous attention to detail, comprising a total of six layers. What makes this model particularly innovative is its ability to process input data, which, in our case, takes the form of a 2D structure that evolves over time. This architecture has yielded remarkable outcomes in our study.

Specifically, the research presented has achieved a balanced accuracy rate of 97.49% for patients diagnosed with ADA and an equally impressive 97.03% for patients with ADM. These results are indicative of the model's robustness and its capacity to accurately distinguish between these two distinct categories of patients. Such high accuracy rates are a testament to the potential of CNN-based models in automating patient classification within medical contexts.

Notably, what sets our study apart from many others in the field is the scale of our dataset. Data from a substantial and diverse database has been offered, incorporating information from a total of five different hospitals. This extensive and multi-hospital dataset is a unique aspect of our research, enhancing the reliability and generalizability of our findings. It underscores the real-world applicability of our proposed model, which can be employed across various healthcare settings for the benefit of patients.

Moreover, it is worth mentioning that our study has delved into the intricacies of the model architecture. The potential for further improving results by increasing the number of convolutional layers has been explored, although acknowledging that this comes at the cost of a more intricate and complex deep structure. This insight opens the door to future research and development, as a fully refined and highly accurate EEG-based diagnostic tool for Alzheimer's disease could have transformative implications within the medical field.

The CNN-based model developed represents an advancement in the realm of Alzheimer's disease detection from EEG data in the time domain. The exceptional accuracy rates achieved, the utilization of a diverse multi-hospital dataset, and the potential for further enhancements through model complexity adjustments all highlight the potential of this research to improve Alzheimer's disease diagnosis and classification. Continued refinement and expansion of these findings bring us closer to the potential development of a sophisticated and precise diagnostic tool with the capacity to benefit the medical community and, ultimately, individuals afflicted by Alzheimer's Disease.

The prospects for Alzheimer's disease detection and classification utilizing CNN-based models are exceptionally promising. The forthcoming research trajectory, stemming from the work presented in this paper, encompasses a range of compelling dimensions. This includes the integration of multimodal data sources to bolster diagnostic accuracy, the execution of longitudinal studies aimed at uncovering early biomarkers, the development of real-time diagnostic tools for swift interventions, the enhancement of model interpretability for more transparent decision-making, and the meticulous conduct of clinical validation studies to ensure robustness. Furthermore, the authors anticipate a global push for accessibility in deploying these advancements in diverse healthcare settings. Moreover, on the horizon lies the endeavor to develop novel neural networks tailored specifically for classification tasks involving ADS versus ADM. This forthcoming work is poised to significantly broaden our comprehension of Alzheimer's disease and its multifaceted presentations, potentially paving the way for more targeted interventions and improved patient care.

## CRediT authorship contribution statement

**Carlos Roncero-Parra:** Writing – review & editing, Writing – original draft, Validation, Software, Methodology, Investigation. **Alfonso Parreño-Torres:** Data curation, Conceptualization. **Roberto Sánchez-Reolid:** Data curation, Conceptualization. **Jorge Mateo-Sotos:** Supervision, Project administration, Methodology, Investigation, Formal analysis. **Alejandro L. Borja:** Writing – review & editing, Writing – original draft, Supervision, Project administration, Methodology, Investigation.

## Declaration of Competing Interest

The authors declare that they have no known competing financial interests or personal relationships that could have appeared to influence the work reported in this paper.

## References

[1] Zeinaband B., Rafik K. (2020). Comprehensive review on Alzheimer's disease: causes and treatment. Molecules.

[2] Yiannopoulou K.G., Papageorgiou S.G. (2020). Current and future treatments in Alzheimer disease: an update. J. Cent. Nerv. Syst. Dis..

[3] Livingston G., Huntley J., Sommerlad A., Ames D., Ballard C., Banerjee S., Brayne C., Mansfield A., Burnsand Cohen-J., Cooper C. (2020). Dementia prevention, intervention, and care: 2020 report of the lancet commission. Lancet.

[4] National institute of neurological disorders and stroke (Jan 2023). Alzheimer's disease. https://www.ninds.nih.gov/health-information/disorders/alzheimers-disease.

[5] Kawas C.H., Kim R.C., Sonnen J.A., Bullain S.S., Trieu T., Corrada M.M. (2015). 2023 Alzheimer's disease facts and figures. Alzheimer's Dement..

[6] Schneider J., Arvanitakis Z., Bang W., Bennett D.A. (2007). Mixed brain pathologies account for most dementia cases in communitydwelling older persons. Neurology.

[7] Sperling R.A., Aisen P.S., Beckett L.A. (2011). Toward defining the pre- clinical stages of Alzheimer's disease: recommendations from the national institute on aging-Alzheimer's association workgroups on diagnostic guidelines for Alzheimer's disease. Alzheimer's Dement..

[8] Jack C.R., Albert M.S., Knopman D.S. (2011). Introduction to the recommendations from the national institute on aging-Alzheimer's association workgroups on diagnostic guidelines for Alzheimer's disease. Alzheimer's Dement..

[9] Vermunt L., Sikkes S., Van den Hout A. (2019). Duration of preclinical, prodromal, and dementia stages of Alzheimer's disease in relation to age, sex, and apoe genotype. Alzheimer's Dement..

[10] (2023). Alzheimer's Dement..

[11] Yupan S., Qinying M.A., Chunyu F., Mingwei W., Hualong W., Bing L., Jiyu F., Shaochen M.A., Xin G., Tongliang L. (2022). Microstate feature fusion for distinguishing AD from MCI. Health Inf. Sci. Syst..

[12] Rajan K.B., Weuve J., Barnes L.L., McAninch E.A., Wilson R.S., Evans D.A. (2021). Population estimate of people with clinical ad and mild cognitive impairment in the United States (2020-2060). Alzheimer's Dement..

[13] Tejada-Vera B. (2013). Mortality from Alzheimer's disease in the United States: data for 2000 and 2010. NCHS Data Brief.

[14] U.S. Department of Health, Human Services, Centers for Disease Control, National Center for Health Statistics Prevention (2022). CDC WONDER online database: about Underlying Cause of Death, 1999-2020. https://www.wonder.cdc.%20gov/ucd-icd10.html.

[15] Dominik K., Fei H., Min W., Matteo D.M., Daniel J.B., Ptolemaios G.S. (2022). Characterising Alzheimer's disease with EEG-based energy landscape analysis. IEEE J. Biomed. Health Inform..

[16] Sinead G., Federico R., Marion H., Marie-Constance C., Lionel N., Jacobo D.S., Bertrand H., Delphine O., Geoffroy G., Marie-Odile H., Bruno D., Fabrizio D.V.F., Hovagim B., Stéphane E. (2019). Eeg evidence of compensatory mechanisms in preclinical Alzheimer's disease. Alzheimer's Dis. Neuroimaging Initiat..

[17] Mohammad-Parsa H., Amin H., Kiarash A. (2021). A review on machine learning for eeg signal processing in bioengineering. IEEE Rev. Biomed. Eng..

[18] Miguel L., María J., Jorge Mateo S., Jorge R., Alejandro B. (2021). A survey on EEG signal processing techniques and machine learning: applications to the neurofeedback of autobiographical memory deficits in schizophrenia. Electronics.

[19] TIBCO Software (2023). Qué es el aprendizaje no supervisado?. https://www.tibco.com/es/reference-center/what-is-unsupervised-learning.

[20] Nichols J.A., Herbert Chan H.W., Baker M.A.B. (2018). Machine learning: applications of artificial intelligence to imaging and diagnosis. Biophys. Rev..

[21] Yu K.H., Beam A.L., Kohane I.S. (2018). Artificial intelligence in healthcare. Nat. Biomed. Eng..

[22] Craik A., He Y., Contreras-Vidal J.L. (2019). Deep learning for electroencephalogram (eeg) classification task. J. Neural Eng..

[23] Gao Z., Dang W., Wang X., Hong X., Hou L., Ma K., Perc M. (2021). Complex networks and deep learning for egg signal analysis. Cogn. Neurodyn..

[24] Robin K. (2012). Modern electroencephalography. J. Neurol..

[25] Burgess R.C. (2019). Filtering of neurophysiologics signals. Handb. Clin. Neurol..

[26] Munday J.A. (2005). Instrumentation and electrode placement. Respir. Care Clin. N. Am..

[27] Niedermeyer Ernst, Lopes da Silva F.H. (2005).

[28] Sreepadmanabh M., Sahu A.K., Chande A. (2020). Covid-19: advances in diagnostic tools. J. Biosci. Treat. Strat. Vaccine Dev..

[29] Kratz A., Lee S.H., Zini G., Riedl J.A., Hur M., Machin S. (2019). International council for standardization in haematology. Digital morphology analyzers in hematology: icsh review and recommendations. Int. J. Lab. Hematol..

[30] Khosrow-Pour D.B.A. (2018).

[31] Rahul P., Jagadeesh P. (2022). International Conference on Business Analytics for Technology and Security (ICBATS).

[32] Punnawish T., Phurin R., Phattarapong S., Phairot A., Rattanaphon C., Nannapas B., Puttaranun B., Nattasate T., Thapanun S., Theerawit W. (2022). Eegwavenet: multiscale cnn-based spatiotemporal feature extraction for eeg seizure detection. IEEE Trans. Ind. Inform..

[33] Salafian B., Ben-Knaan E.F., Shlezinger N., De-Ribaupierre S., Farsad N. (2023). Mical: mutual information-based cnn-aided learned factor graphs for seizure detection from eeg signals. IEEE Access.

[34] Amin S.U., Alsulaiman M., Muhammad G., Bencherif M.A., Hossain M.S. (2019). Multilevel weighted feature fusion using convolutional neural networks for eeg motor imagery classification. IEEE Access.

[35] Utz H.F., Melchinger A.E., Schön C.C. (2000). Bias and sampling error of the estimated proportion of genotypic variance explained by quantitative trait loci determined from experimental data in maize using cross validation and validation with independent samples. Genetics.

[36] Moher D., Liberati A., Tetzlaff J., Altman D.G. (2009). Preferred reporting items for systematic reviews and meta-analyses: the prisma statement. J. Clin. Epidemiol..

[37] Georgios G., Konstantina L., Konstantinos D., Ioanna C., Magda T. (2022). Diagnosis of Alzheimer's disease and mild cognitive impairment using eeg and recurrent neural networks. IEEE Eng. Med. Biol. Soc..

[38] Wei X., Ran Z., Xiao Z., Muhammad U. (2023). A novel method for diagnosing Alzheimer's disease using deep pyramid cnn based on eeg signals. Heliyon.

[39] Perez-Valero E., Morillas C., Lopez-Gordo M.A., Carrera-Muñoz I., López-Alcalde S., Vílchez-Carrillo R.M. (2022). An automated approach for the detection of Alzheimer's disease from resting state electroencephalography. Front. Neuroinform..

[40] Lopes M., Cassani R., Falk T.H. (2023). Using cnn saliency maps and eeg modulation spectra for improved and more interpretable machine learning-based Alzheimer's disease diagnosis. Comput. Intell. Neurosci..

[41] AlSharabi K., Bin-Salamah Y., Abdurraqeeb A.M., Aljalal M., Alturki F.A. (2022). Eeg signal processing for Alzheimer's disorders using discrete wavelet transform and machine learning approaches. IEEE Access.

[42] Khatun S., Morshed B.I., Bidelman G.M. (2019). A single-channel eeg-based approach to detect mild cognitive impairment via speech-evoked brain responses. IEEE Trans. Neural Syst. Rehabil. Eng..

